# Supplementary figures and images for: Interpretable machine learning models to predict short-term postoperative outcomes following posterior cervical fusion
Source: PLoS One. 2023 Jul 21;18(7):e0288939. doi: 10.1371/journal.pone.0288939 (PMC10361477; doi:10.1371/journal.pone.0288939)

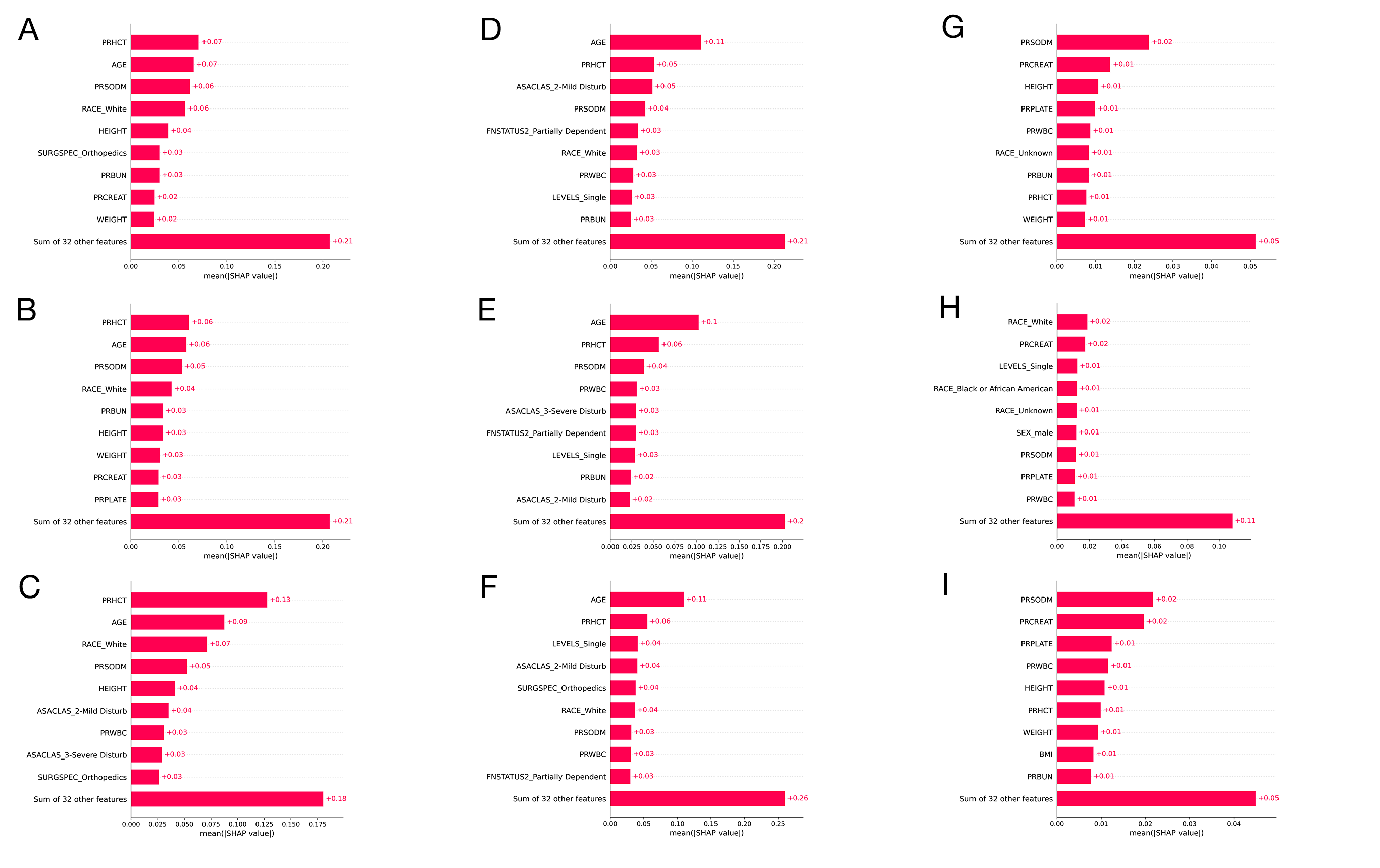

Supplement: S1 Fig — A) The ten most important features and their mean SHAP values for the model predicting prolonged length of stay with the XGBoost algorithm, B) the ten most important features and their mean SHAP values for the model predicting prolonged length of stay with the LightGBM algorithm, C) the ten most important features and their mean SHAP values for the model predicting prolonged length of stay with the Random Forest algorithm, D) the ten most important features and their mean SHAP values for the model predicting nonhome discharges with the XGBoost algorithm, E) the ten most important features and their mean SHAP values for the model predicting nonhome discharges with the LightGBM algorithm, F) the ten most important features and their mean SHAP values for the model predicting nonhome discharges with the CatBoost algorithm, G) the ten most important features and their mean SHAP values for the model predicting readmissions with the XGBoost algorithm, H) the ten most important features and their mean SHAP values for the model predicting readmissions with the CatBoost algorithm, I) the ten most important features and their mean SHAP values for the model predicting readmissions with the Random Forest algorithm. (TIF) [file pone.0288939.s002.tif]
