# Supplementary material for: Interpretable machine learning models to predict short-term postoperative outcomes following posterior cervical fusion
Source: PLoS One. 2023 Jul 21;18(7):e0288939. doi: 10.1371/journal.pone.0288939 (PMC10361477; doi:10.1371/journal.pone.0288939)
Supplement: S1 Table — (DOCX) [file pone.0288939.s003.docx]

**S1 Table.** CPT codes that were used to exclude patients.

|  | **CPT** | **CPT description** |
| --- | --- | --- |
| **Anterior cervical procedures** | 63075 | Discectomy, anterior, with decompression of spinal cord and/or nerve root(s), including osteophytectomy; cervical, single interspace |
|  | 22554 | Arthrodesis, anterior interbody technique, including minimal discectomy to prepare interspace (other than for decompression); cervical below C2 |
|  | 22585 | Arthrodesis, anterior interbody technique, including minimal discectomy to prepare interspace (other than for decompression); each additional interspace |
|  | 22551 | Arthrodesis, anterior interbody, including disc space preparation, discectomy, osteophytectomy and decompression of spinal cord and/or nerve roots; cervical below C2 |
|  | 22552 | Arthrodesis, anterior interbody, including disc space preparation, discectomy, osteophytectomy and decompression of spinal cord and/or nerve roots; cervical below C2, each additional interspace |
|  | 22220 | Osteotomy of spine, including discectomy, anterior approach, single vertebral segment; cervical |
| **Thoracic and lumbar fusion** | 22556 | Arthrodesis, anterior interbody, including disc space preparation, discectomy, osteophytectomy and decompression of spinal cord and/or nerve roots; thoracic |
|  | 22558 | Arthrodesis, anterior interbody, including disc space preparation, discectomy, osteophytectomy and decompression of spinal cord and/or nerve roots; lumbar |
|  | 22610 | Arthrodesis, posterior or posterolateral technique, single level; thoracic |
|  | 22612 | Arthrodesis, posterior or posterolateral technique, single level; lumbar |
|  | 22630 | Arthrodesis, posterior or posterolateral technique, including laminectomy and/or discectomy to prepare interspace (other than for decompression), single interspace; lumbar |
|  | 22632 | Arthrodesis, posterior or posterolateral technique, including laminectomy and/or discectomy to prepare interspace (other than for decompression), single interspace; each additional interspace |
|  | 22633 | Arthrodesis, combined posterior or posterolateral technique with posterior interbody technique including laminectomy and/or discectomy sufficient to prepare interspace (other than for decompression), single interspace and segment; lumbar |
|  | 22634 | Arthrodesis, combined posterior or posterolateral technique with posterior interbody technique including laminectomy and/or discectomy sufficient to prepare interspace (other than for decompression), single interspace and segment; each additional interspace and segment |
| **Revision** | 22830 | Exploration of spinal fusion |
|  | 22849 | Reinsertion of spinal fixation device |
|  | 22850 | Removal of posterior nonsegmental instrumentation |
|  | 22852 | Removal of posterior segmental instrumentation |
|  | 22855 | Removal of anterior instrumentation |
| **Intraspinal lesion** | 63300 | Vertebral corpectomy (vertebral body resection), partial or complete, for excision of intraspinal lesion, single segment; extradural, cervical |
|  | 63301 | Vertebral corpectomy (vertebral body resection), partial or complete, for excision of intraspinal lesion, single segment; extradural, thoracic by transthoracic approach |
|  | 63304 | Vertebral corpectomy (vertebral body resection), partial or complete, for excision of intraspinal lesion, single segment; intradural, cervical |
|  | 63308 | Vertebral corpectomy (vertebral body resection), partial or complete, for excision of intraspinal lesion, single segment; each additional segment |
