# Supplementary material for: Interpretable machine learning models to predict short-term postoperative outcomes following posterior cervical fusion
Source: PLoS One. 2023 Jul 21;18(7):e0288939. doi: 10.1371/journal.pone.0288939 (PMC10361477; doi:10.1371/journal.pone.0288939)
Supplement: S2 Table — (DOCX) [file pone.0288939.s004.docx]

**S2 Table.** Characteristics of the patient population, both among the non-prolonged LOS and prolonged LOS groups and in total.

| **Variables** | | **No Prolonged Lenght of Stay** (n=4807) | **Prolonged Length of Stay** (n=1470) | **p Values** | **Total** |
| --- | --- | --- | --- | --- | --- |
|  |  | Mean (±SD), Median (IQR), or n (%) | |  | Mean (±SD), Median (IQR), or n (%) |
| **Age** | | 61.0 (15.0) | 65.0 (16.0) | <0.001 | 62.0 (15.0) |
| **Sex** | **Female** | 2119 (44.1%) | 680 (46.3%) | 0.15 | 2799 (44.6%) |
|  | **Male** | 2688 (55.9%) | 790 (53.7%) |  | 3478 (55.4%) |
| **Race/Ethnicity** | **Asian** | 68 (1.4%) | 25 (1.7%) | <0.001 | 93 (1.5%) |
|  | **Black or African American** | 634 (13.2%) | 277 (18.8%) |  | 911 (14.5%) |
|  | **Hispanic** | 265 (5.5%) | 100 (6.8%) |  | 365 (5.8%) |
|  | **Other** | 51 (1.1%) | 32 (2.2%) |  | 83 (1.3%) |
|  | **Unknown** | 300 (6.2%) | 164 (11.2%) |  | 464 (7.4%) |
|  | **White** | 3489 (72.6%) | 872 (59.3%) |  | 4361 (69.5%) |
| **BMI** | | 29.34 (7.65) | 29.11 (8.31) | 0.936 | 29.29 (7.92) |
| **Transfer Status** | **Not transferred** | 4768 (99.2%) | 1405 (95.6%) | <0.001 | 6173 (98.3%) |
|  | **Transferred** | 37 (0.8%) | 64 (4.4%) |  | 101 (1.6%) |
|  | **Unknown** | 2 (0.0%) | 1 (0.1%) |  | 3 (0.0%) |
| **Diabetes** | **No** | 3863 (80.4%) | 1064 (72.4%) | <0.001 | 4927 (78.5%) |
|  | **Yes** | 944 (19.6%) | 406 (27.6%) |  | 1350 (21.5%) |
| **Smoker Within 1 Year** | **No** | 3646 (75.8%) | 1119 (76.1%) | 0.857 | 4765 (75.9%) |
|  | **Yes** | 1161 (24.2%) | 351 (23.9%) |  | 1512 (24.1%) |
| **Dyspnea** | **No** | 4529 (94.2%) | 1355 (92.2%) | 0.006 | 5884 (93.7%) |
|  | **Yes** | 278 (5.8%) | 115 (7.8%) |  | 393 (6.3%) |
| **Ventilator Dependency** | **No** | 4806 (100.0%) | 1469 (99.9%) | 0.958 | 6275 (100.0%) |
|  | **Yes** | 1 (0.0%) | 1 (0.1%) |  | 2 (0.0%) |
| **History of Severe COPD** | **No** | 4495 (93.5%) | 1331 (90.5%) | <0.001 | 5826 (92.8%) |
|  | **Yes** | 312 (6.5%) | 139 (9.5%) |  | 451 (7.2%) |
| **Congestive Heart Failure Within 30 Days Prior to Surgery** | **No** | 4786 (99.6%) | 1456 (99.0%) | 0.034 | 6242 (99.4%) |
|  | **Yes** | 21 (0.4%) | 14 (1.0%) |  | 35 (0.6%) |
| **Hypertension Requiring Medication** | **No** | 2066 (43.0%) | 499 (34.0%) | <0.001 | 2565 (40.9%) |
|  | **Yes** | 2741 (57.0%) | 971 (66.0%) |  | 3712 (59.1%) |
| **Acute Renal Failure** | **No** | 4805 (100.0%) | 1464 (99.6%) | 0.002 | 6269 (99.9%) |
|  | **Yes** | 2 (0.0%) | 6 (0.4%) |  | 8 (0.1%) |
| **Currently Requiring or On Dialysis** | **No** | 4797 (99.8%) | 1456 (99.0%) | <0.001 | 6253 (99.6%) |
|  | **Yes** | 10 (0.2%) | 14 (1.0%) |  | 24 (0.4%) |
| **Disseminated Cancer** | **No** | 4803 (99.9%) | 1462 (99.5%) | 0.001 | 6265 (99.8%) |
|  | **Yes** | 4 (0.1%) | 8 (0.5%) |  | 12 (0.2%) |
| **Steroid or Immunosuppressant for a Chronic Condition** | **No** | 4583 (95.3%) | 1394 (94.8%) | 0.464 | 5977 (95.2%) |
|  | **Yes** | 224 (4.7%) | 76 (5.2%) |  | 300 (4.8%) |
| **>10% Loss of Body Weight in last 6 months** | **No** | 4797 (99.8%) | 1456 (99.0%) | <0.001 | 6253 (99.6%) |
|  | **Yes** | 10 (0.2%) | 14 (1.0%) |  | 24 (0.4%) |
| **Bleeding Disorders** | **No** | 4739 (98.6%) | 1435 (97.6%) | 0.015 | 6174 (98.4%) |
|  | **Yes** | 68 (1.4%) | 35 (2.4%) |  | 103 (1.6%) |
| **Pre-Operative RBC Transfusion** | **No** | 4805 (100.0%) | 1468 (99.9%) | 0.506 | 6273 (99.9%) |
|  | **Yes** | 2 (0.0%) | 2 (0.1%) |  | 4 (0.1%) |
| **Wound Infection** | **No** | 4795 (99.8%) | 1448 (98.5%) | <0.001 | 6243 (99.5%) |
|  | **Yes** | 12 (0.2%) | 22 (1.5%) |  | 34 (0.5%) |
| **ASA Classification** | **1 (No Disturb)** | 45 (0.9%) | 4 (0.3%) | <0.001 | 49 (0.8%) |
|  | **2 (Mild Disturb)** | 1892 (39.4%) | 377 (25.6%) |  | 2269 (36.2%) |
|  | **3 (Severe Disturb)** | 2870 (59.7%) | 1089 (74.1%) |  | 3959 (63.1%) |
| **Functional Status Prior to Surgery** | **Independent** | 4664 (97.0%) | 1345 (91.5%) | <0.001 | 6009 (95.7%) |
|  | **Partially Dependent** | 105 (2.2%) | 107 (7.3%) |  | 212 (3.4%) |
|  | **Totally Dependent** | 18 (0.4%) | 13 (0.9%) |  | 31 (0.5%) |
|  | **Unknown** | 20 (0.4%) | 5 (0.3%) |  | 25 (0.4%) |
| **Inpatient or Outpatient** | **Inpatient** | 140.0 (3.0) | 139.0 (3.0) | <0.001 | 140.0 (3.0) |
|  | **Outpatient** | 16.0 (±7.0) | 16.0 (8.13) | 0.007 | 16.0 (±7.2) |
| **Serum Sodium** | | 0.89 (±0.26) | 0.89 (±0.31) | 0.7 | 0.89 (±0.27) |
| **Serum BUN** | | 7.1 (±2.7) | 7.0 (2.9) | 0.873 | 7.1 (±2.7) |
| **Serum Creatinine** | | 41.7 (5.3) | 40.3 (6.0) | <0.001 | 41.3 (5.5) |
| **White Blood Cell Count** | | 241.0 (86.0) | 239.0 (84.75) | 0.128 | 241.0 (85.0) |
| **Hematocrit** | | 4471 (93.0%) | 1451 (98.7%) | <0.001 | 5922 (94.3%) |
| **Platelet Count** | | 336 (7.0%) | 19 (1.3%) |  | 355 (5.7%) |
| **Surgical Specialty** | **Neurosurgery** | 2487 (51.7%) | 835 (56.8%) | 0.001 | 3322 (52.9%) |
|  | **Orthopedics** | 2320 (48.3%) | 635 (43.2%) |  | 2955 (47.1%) |
| **Fusion Levels** | **Multi** | 3352 (69.7%) | 1128 (76.7%) | <0.001 | 4480 (71.4%) |
|  | **Single** | 1455 (30.3%) | 342 (23.3%) |  | 1797 (28.6%) |
| **Non-home Discharge** | **No** | 4195 (87.3%) | 793 (54.0%) | <0.001 | 4988 (79.5%) |
|  | **Yes** | 612 (12.7%) | 677 (46.0%) |  | 1289 (20.5%) |
| **Readmission** | **No** | 4489 (93.4%) | 1363 (92.7%) | 0.408 | 5852 (93.2%) |
|  | **Yes** | 318 (6.6%) | 107 (7.3%) |  | 425 (6.8%) |
