# Supplementary material for: Interpretable machine learning models to predict short-term postoperative outcomes following posterior cervical fusion
Source: PLoS One. 2023 Jul 21;18(7):e0288939. doi: 10.1371/journal.pone.0288939 (PMC10361477; doi:10.1371/journal.pone.0288939)
Supplement: S3 Table — (DOCX) [file pone.0288939.s005.docx]

**S3 Table.** Characteristics of the patient population, both among the home discharge and nonhome discharge groups and in total.

| **Variables** | | **Home Discharge** (n=4988) | **Non-home Discharge** (n=1289) | **p Values** | **Total** |
| --- | --- | --- | --- | --- | --- |
|  |  | Mean (±SD), Median (IQR), or n (%) | |  | Mean (±SD), Median (IQR), or n (%) |
| **Age** | | 61.0 (15.0) | 68.0 (14.0) | <0.001 | 62.0 (15.0) |
| **Sex** | **Female** | 2213 (44.4%) | 586 (45.5%) | 0.501 | 2799 (44.6%) |
|  | **Male** | 2775 (55.6%) | 703 (54.5%) |  | 3478 (55.4%) |
| **Race/Ethnicity** | **Asian** | 62 (1.2%) | 31 (2.4%) | <0.001 | 93 (1.5%) |
|  | **Black or African American** | 649 (13.0%) | 262 (20.3%) |  | 911 (14.5%) |
|  | **Hispanic** | 271 (5.4%) | 94 (7.3%) |  | 365 (5.8%) |
|  | **Other** | 74 (1.5%) | 9 (0.7%) |  | 83 (1.3%) |
|  | **Unknown** | 384 (7.7%) | 80 (6.2%) |  | 464 (7.4%) |
|  | **White** | 3548 (71.1%) | 813 (63.1%) |  | 4361 (69.5%) |
| **BMI** | | 29.35 (7.75) | 29.1 (8.24) | 0.619 | 29.29 (7.92) |
| **Transfer Status** | **Not transferred** | 4950 (99.2%) | 1223 (94.9%) | <0.001 | 6173 (98.3%) |
|  | **Transferred** | 36 (0.7%) | 65 (5.0%) |  | 101 (1.6%) |
|  | **Unknown** | 2 (0.0%) | 1 (0.1%) |  | 3 (0.0%) |
| **Diabetes** | **No** | 4024 (80.7%) | 903 (70.0%) | <0.001 | 4927 (78.5%) |
|  | **Yes** | 964 (19.3%) | 386 (30.0%) |  | 1350 (21.5%) |
| **Smoker Within 1 Year** | **No** | 3741 (75.0%) | 1024 (79.4%) | 0.001 | 4765 (75.9%) |
|  | **Yes** | 1247 (25.0%) | 265 (20.6%) |  | 1512 (24.1%) |
| **Dyspnea** | **No** | 4709 (94.4%) | 1175 (91.2%) | <0.001 | 5884 (93.7%) |
|  | **Yes** | 279 (5.6%) | 114 (8.8%) |  | 393 (6.3%) |
| **Ventilator Dependency** | **No** | 4987 (100.0%) | 1288 (99.9%) | 0.876 | 6275 (100.0%) |
|  | **Yes** | 1 (0.0%) | 1 (0.1%) |  | 2 (0.0%) |
| **History of Severe COPD** | **No** | 4666 (93.5%) | 1160 (90.0%) | <0.001 | 5826 (92.8%) |
|  | **Yes** | 322 (6.5%) | 129 (10.0%) |  | 451 (7.2%) |
| **Congestive Heart Failure Within 30 Days Prior to Surgery** | **No** | 4968 (99.6%) | 1274 (98.8%) | 0.002 | 6242 (99.4%) |
|  | **Yes** | 20 (0.4%) | 15 (1.2%) |  | 35 (0.6%) |
| **Hypertension Requiring Medication** | **No** | 2180 (43.7%) | 385 (29.9%) | <0.001 | 2565 (40.9%) |
|  | **Yes** | 2808 (56.3%) | 904 (70.1%) |  | 3712 (59.1%) |
| **Acute Renal Failure** | **No** | 4981 (99.9%) | 1288 (99.9%) | 0.9 | 6269 (99.9%) |
|  | **Yes** | 7 (0.1%) | 1 (0.1%) |  | 8 (0.1%) |
| **Currently Requiring or On Dialysis** | **No** | 4974 (99.7%) | 1279 (99.2%) | 0.021 | 6253 (99.6%) |
|  | **Yes** | 14 (0.3%) | 10 (0.8%) |  | 24 (0.4%) |
| **Disseminated Cancer** | **No** | 4978 (99.8%) | 1287 (99.8%) | 1.0 | 6265 (99.8%) |
|  | **Yes** | 10 (0.2%) | 2 (0.2%) |  | 12 (0.2%) |
| **Steroid or Immunosuppressant for a Chronic Condition** | **No** | 4764 (95.5%) | 1213 (94.1%) | 0.042 | 5977 (95.2%) |
|  | **Yes** | 224 (4.5%) | 76 (5.9%) |  | 300 (4.8%) |
| **>10% Loss of Body Weight in last 6 months** | **No** | 4981 (99.9%) | 1272 (98.7%) | <0.001 | 6253 (99.6%) |
|  | **Yes** | 7 (0.1%) | 17 (1.3%) |  | 24 (0.4%) |
| **Bleeding Disorders** | **No** | 4919 (98.6%) | 1255 (97.4%) | 0.002 | 6174 (98.4%) |
|  | **Yes** | 69 (1.4%) | 34 (2.6%) |  | 103 (1.6%) |
| **Pre-Operative RBC Transfusion** | **No** | 4988 (100.0%) | 1285 (99.7%) | 0.001 | 6273 (99.9%) |
|  | **Yes** | 0 (0.0%) | 4 (0.3%) |  | 4 (0.1%) |
| **Wound Infection** | **No** | 4976 (99.8%) | 1267 (98.3%) | <0.001 | 6243 (99.5%) |
|  | **Yes** | 12 (0.2%) | 22 (1.7%) |  | 34 (0.5%) |
| **ASA Classification** | **1 (No Disturb)** | 49 (1.0%) | 0 (0.0%) | <0.001 | 49 (0.8%) |
|  | **2 (Mild Disturb)** | 2018 (40.5%) | 251 (19.5%) |  | 2269 (36.2%) |
|  | **3 (Severe Disturb)** | 2921 (58.6%) | 1038 (80.5%) |  | 3959 (63.1%) |
| **Functional Status Prior to Surgery** | **Independent** | 4885 (97.9%) | 1124 (87.2%) | <0.001 | 6009 (95.7%) |
|  | **Partially Dependent** | 71 (1.4%) | 141 (10.9%) |  | 212 (3.4%) |
|  | **Totally Dependent** | 14 (0.3%) | 17 (1.3%) |  | 31 (0.5%) |
|  | **Unknown** | 18 (0.4%) | 7 (0.5%) |  | 25 (0.4%) |
| **Inpatient or Outpatient** | **Inpatient** | 140.0 (3.0) | 140.0 (3.0) | 0.133 | 140.0 (3.0) |
|  | **Outpatient** | 15.2 (±7.0) | 17.0 (9.0) | <0.001 | 16.0 (±7.2) |
| **Serum Sodium** | | 0.89 (±0.26) | 0.9 (±0.33) | 0.005 | 0.89 (±0.27) |
| **Serum BUN** | | 7.1 (±2.7) | 7.0 (2.9) | 0.996 | 7.1 (±2.7) |
| **Serum Creatinine** | | 41.78 (5.3) | 40.0 (5.8) | <0.001 | 41.3 (5.5) |
| **White Blood Cell Count** | | 242.0 (86.0) | 236.0 (83.0) | <0.001 | 241.0 (85.0) |
| **Hematocrit** | | 4651 (93.2%) | 1271 (98.6%) | <0.001 | 5922 (94.3%) |
| **Platelet Count** | | 337 (6.8%) | 18 (1.4%) |  | 355 (5.7%) |
| **Surgical Specialty** | **Neurosurgery** | 2552 (51.2%) | 770 (59.7%) | <0.001 | 3322 (52.9%) |
|  | **Orthopedics** | 2436 (48.8%) | 519 (40.3%) |  | 2955 (47.1%) |
| **Fusion Levels** | **Multi** | 3505 (70.3%) | 975 (75.6%) | <0.001 | 4480 (71.4%) |
|  | **Single** | 1483 (29.7%) | 314 (24.4%) |  | 1797 (28.6%) |
| **Prolonged Length of Stay** | **No** | 4195 (84.1%) | 612 (47.5%) | <0.001 | 4988 (79.5%) |
|  | **Yes** | 793 (15.9%) | 677 (52.5%) |  | 1289 (20.5%) |
| **Readmission** | **No** | 4656 (93.3%) | 1196 (92.8%) | 0.516 | 5852 (93.2%) |
|  | **Yes** | 332 (6.7%) | 93 (7.2%) |  | 425 (6.8%) |
