# Supplementary material for: Interpretable machine learning models to predict short-term postoperative outcomes following posterior cervical fusion
Source: PLoS One. 2023 Jul 21;18(7):e0288939. doi: 10.1371/journal.pone.0288939 (PMC10361477; doi:10.1371/journal.pone.0288939)
Supplement: S4 Table — (DOCX) [file pone.0288939.s006.docx]

**S4 Table.** Characteristics of the patient population, both among the no readmission and readmission groups and in total.

| **Variables** | | **No Readmission** (n=5852) | **Readmission** (n=425) | **p Values** | **Total** |
| --- | --- | --- | --- | --- | --- |
|  |  | Mean (±SD), Median (IQR), or n (%) | |  | Mean (±SD), Median (IQR), or n (%) |
| **Age** | | 62.0 (16.0) | 64.0 (15.0) | 0.011 | 62.0 (15.0) |
| **Sex** | **Female** | 2638 (45.1%) | 161 (37.9%) | 0.005 | 2799 (44.6%) |
|  | **Male** | 3214 (54.9%) | 264 (62.1%) |  | 3478 (55.4%) |
| **Race/Ethnicity** | **Asian** | 83 (1.4%) | 10 (2.4%) | 0.019 | 93 (1.5%) |
|  | **Black or African American** | 833 (14.2%) | 78 (18.4%) |  | 911 (14.5%) |
|  | **Hispanic** | 343 (5.9%) | 22 (5.2%) |  | 365 (5.8%) |
|  | **Other** | 77 (1.3%) | 6 (1.4%) |  | 83 (1.3%) |
|  | **Unknown** | 446 (7.6%) | 18 (4.2%) |  | 464 (7.4%) |
|  | **White** | 4070 (69.6%) | 291 (68.5%) |  | 4361 (69.5%) |
| **BMI** | | 29.21 (7.87) | 30.13 (7.92) | 0.032 | 29.29 (7.92) |
| **Transfer Status** | **Not transferred** | 5754 (98.3%) | 419 (98.6%) | 0.848 | 6173 (98.3%) |
|  | **Transferred** | 95 (1.6%) | 6 (1.4%) |  | 101 (1.6%) |
|  | **Unknown** | 3 (0.0%) | 0 (0.0%) |  | 3 (0.0%) |
| **Diabetes** | **No** | 4629 (79.1%) | 298 (70.1%) | <0.001 | 4927 (78.5%) |
|  | **Yes** | 1223 (20.9%) | 127 (29.9%) |  | 1350 (21.5%) |
| **Smoker Within 1 Year** | **No** | 4446 (76.0%) | 319 (75.1%) | 0.713 | 4765 (75.9%) |
|  | **Yes** | 1406 (24.0%) | 106 (24.9%) |  | 1512 (24.1%) |
| **Dyspnea** | **No** | 5502 (94.0%) | 382 (89.9%) | 0.001 | 5884 (93.7%) |
|  | **Yes** | 350 (6.0%) | 43 (10.1%) |  | 393 (6.3%) |
| **Ventilator Dependency** | **No** | 5850 (100.0%) | 425 (100.0%) | 1.0 | 6275 (100.0%) |
|  | **Yes** | 2 (0.0%) | 0 (0.0%) |  | 2 (0.0%) |
| **History of Severe COPD** | **No** | 5446 (93.1%) | 380 (89.4%) | 0.007 | 5826 (92.8%) |
|  | **Yes** | 406 (6.9%) | 45 (10.6%) |  | 451 (7.2%) |
| **Congestive Heart Failure Within 30 Days Prior to Surgery** | **No** | 5820 (99.4%) | 422 (99.3%) | 0.93 | 6242 (99.4%) |
|  | **Yes** | 32 (0.6%) | 3 (0.7%) |  | 35 (0.6%) |
| **Hypertension Requiring Medication** | **No** | 2435 (41.6%) | 130 (30.6%) | <0.001 | 2565 (40.9%) |
|  | **Yes** | 3417 (58.4%) | 295 (69.4%) |  | 3712 (59.1%) |
| **Acute Renal Failure** | **No** | 5845 (99.9%) | 424 (99.8%) | 1.0 | 6269 (99.9%) |
|  | **Yes** | 7 (0.1%) | 1 (0.2%) |  | 8 (0.1%) |
| **Currently Requiring or On Dialysis** | **No** | 5835 (99.7%) | 418 (98.4%) | <0.001 | 6253 (99.6%) |
|  | **Yes** | 17 (0.3%) | 7 (1.6%) |  | 24 (0.4%) |
| **Disseminated Cancer** | **No** | 5841 (99.8%) | 424 (99.8%) | 1.0 | 6265 (99.8%) |
|  | **Yes** | 11 (0.2%) | 1 (0.2%) |  | 12 (0.2%) |
| **Steroid or Immunosuppressant for a Chronic Condition** | **No** | 5579 (95.3%) | 398 (93.6%) | 0.145 | 5977 (95.2%) |
|  | **Yes** | 273 (4.7%) | 27 (6.4%) |  | 300 (4.8%) |
| **>10% Loss of Body Weight in last 6 months** | **No** | 5829 (99.6%) | 424 (99.8%) | 0.919 | 6253 (99.6%) |
|  | **Yes** | 23 (0.4%) | 1 (0.2%) |  | 24 (0.4%) |
| **Bleeding Disorders** | **No** | 5757 (98.4%) | 417 (98.1%) | 0.835 | 6174 (98.4%) |
|  | **Yes** | 95 (1.6%) | 8 (1.9%) |  | 103 (1.6%) |
| **Pre-Operative RBC Transfusion** | **No** | 5848 (99.9%) | 425 (100.0%) | 1.0 | 6273 (99.9%) |
|  | **Yes** | 4 (0.1%) | 0 (0.0%) |  | 4 (0.1%) |
| **Wound Infection** | **No** | 5821 (99.5%) | 422 (99.3%) | 0.892 | 6243 (99.5%) |
|  | **Yes** | 31 (0.5%) | 3 (0.7%) |  | 34 (0.5%) |
| **ASA Classification** | **1 (No Disturb)** | 48 (0.8%) | 1 (0.2%) | 0.078 | 49 (0.8%) |
|  | **2 (Mild Disturb)** | 2132 (36.4%) | 137 (32.2%) |  | 2269 (36.2%) |
|  | **3 (Severe Disturb)** | 3672 (62.8%) | 287 (67.5%) |  | 3959 (63.1%) |
| **Functional Status Prior to Surgery** | **Independent** | 5611 (95.9%) | 398 (93.6%) | 0.071 | 6009 (95.7%) |
|  | **Partially Dependent** | 193 (3.3%) | 19 (4.5%) |  | 212 (3.4%) |
|  | **Totally Dependent** | 27 (0.5%) | 4 (0.9%) |  | 31 (0.5%) |
|  | **Unknown** | 21 (0.4%) | 4 (0.9%) |  | 25 (0.4%) |
| **Inpatient or Outpatient** | **Inpatient** | 140.0 (3.0) | 139.8 (3.0) | 0.558 | 140.0 (3.0) |
|  | **Outpatient** | 16.0 (±7.0) | 16.6 (8.0) | 0.001 | 16.0 (±7.2) |
| **Serum Sodium** | | 0.89 (±0.27) | 0.95 (0.37) | <0.001 | 0.89 (±0.27) |
| **Serum BUN** | | 7.1 (±2.7) | 7.1 (2.9) | 0.848 | 7.1 (±2.7) |
| **Serum Creatinine** | | 41.3 (5.4) | 41.38 (±4.78) | 0.739 | 41.3 (5.5) |
| **White Blood Cell Count** | | 241.0 (85.0) | 242.8 (87.0) | 0.048 | 241.0 (85.0) |
| **Hematocrit** | | 5510 (94.2%) | 412 (96.9%) | 0.022 | 5922 (94.3%) |
| **Platelet Count** | | 342 (5.8%) | 13 (3.1%) |  | 355 (5.7%) |
| **Surgical Specialty** | **Neurosurgery** | 3063 (52.3%) | 259 (60.9%) | 0.001 | 3322 (52.9%) |
|  | **Orthopedics** | 2789 (47.7%) | 166 (39.1%) |  | 2955 (47.1%) |
| **Fusion Levels** | **Multi** | 4170 (71.3%) | 310 (72.9%) | 0.493 | 4480 (71.4%) |
|  | **Single** | 1682 (28.7%) | 115 (27.1%) |  | 1797 (28.6%) |
| **Prolonged Length of Stay** | **No** | 4489 (76.7%) | 318 (74.8%) | 0.408 | 4988 (79.5%) |
|  | **Yes** | 1363 (23.3%) | 107 (25.2%) |  | 1289 (20.5%) |
| **Non-home Discharge** | **No** | 4656 (79.6%) | 332 (78.1%) | 0.516 | 4988 (79.5%) |
|  | **Yes** | 1196 (20.4%) | 93 (21.9%) |  | 1289 (20.5%) |
