# Supplementary material for: Interpretable machine learning models to predict short-term postoperative outcomes following posterior cervical fusion
Source: PLoS One. 2023 Jul 21;18(7):e0288939. doi: 10.1371/journal.pone.0288939 (PMC10361477; doi:10.1371/journal.pone.0288939)
Supplement: Attachment — Submitted filename: Response_to_Reviewers.docx [file pone.0288939.s007.docx]

**Response to Reviewers**

**Additional Editor Comments**

References were updated as directed.

Figures were uploaded.
